# Prevalence of second mesiobuccal canal in maxillary first and second molar detected using cone-beam computed tomography in the Han population: A systemic review and meta-analysis

**DOI:** 10.1016/j.jds.2025.09.008

**Published:** 2025-09-22

**Authors:** Jaan-Yih Wu, Ho-Sheng Chiang, Yi-Jie Chen, Zi-Jun Dai, Yen-Ching Chao, Hsu-Hua Chu, Yi-Shing Shieh, Yu-Chiao Wu

**Affiliations:** a Department of Operative Dentistry and Endodontics, College of Oral Medicine, Tri-Service General Hospital and National Defense Medical University, Taipei, Taiwan; b Department and Graduate Institute of Biochemistry, National Defense Medical University, Taipei, Taiwan; c Department of Risk Management and Insurance, National Chengchi University, Taipei, Taiwan

**Keywords:** Cone-beam computed tomography, Han population, Maxilla, Molar, Second mesiobuccal canal

## Abstract

**Background/purpose:**

A thorough understanding of root canal morphology, particularly the second mesiobuccal (MB2) canal, is crucial for successful endodontic treatment of maxillary molars. The prevalence of MB2 shows ethnic variability, and this systematic review and meta-analysis aimed to synthesize existing CBCT-based studies to determine the prevalence of the second mesiobuccal canal (MB2) in the Han population.

**Materials and methods:**

A systematic review and meta-analysis were conducted in accordance with the Preferred Reporting Items for Systematic Reviews and Meta-Analyses (PRISMA) guidelines. Databases including PubMed, MEDLINE, Scopus, and ClinicalKey were searched through 17th August 2025. Studies reporting MB2 prevalence in permanent maxillary first and/or second molars within Han populations using CBCT were included. Data were pooled using a random-effects model.

**Results:**

Fourteen eligible studies involving 15,639 participants and 35,929 teeth were analyzed. The pooled MB2 prevalence was 63.7 % for permanent maxillary first molars (PMFMs) and 23.3 % for permanent maxillary second molars (PMSMs). Males had significantly higher odds of having MB2 canals in both molar types (Odds Ratio = 1.532 and 1.790, respectively). No significant difference was found between left and right sides. Heterogeneity was high (*I*^*2*^ > 95 %), but most studies were of high methodological quality.

**Conclusion:**

MB2 canals are common in the Han population, particularly in PMFMs. Clinicians may consider advanced imaging like CBCT to improve detection and treatment outcomes. Sex-related differences further underscore the need for individualized diagnostic approaches.

## Introduction

A thorough understanding of root canal anatomy is essential for achieving consistent and successful outcomes in endodontic treatment. The permanent maxillary first and second molars are particularly known for their anatomical complexity, especially due to variations such as the presence of a second mesiobuccal canal (MB2).^1^ The permanent maxillary first molars (PMFMs) have been extensively investigated because of their higher prevalence of MB2 canals. In contrast, the maxillary second molars also exhibit significant morphological diversity, including root fusion, C-shaped canal configurations, and atypical numbers of roots.^2^ The frequency of MB2 canals in permanent maxillary second molars (PMSMs) is therefore of notable clinical importance. Failure to detect all canals, particularly MB2, remains a common cause of persistent periapical pathology, microbial persistence, and endodontic failure. When clinicians overlook or inadequately treat these canals, residual infected tissues may remain in the root canal system, potentially leading to apical periodontitis.^3^

Given these challenges, a thorough understanding of the internal anatomy and its variations is crucial for effective diagnosis, access cavity design, canal negotiation, and complete chemomechanical debridement. Advances in diagnostic tools such as cone-beam computed tomography (CBCT) have significantly improved clinicians’ ability to visualize and interpret complex canal configurations.^4^ By incorporating a detailed understanding of root canal morphology into clinical practice, endodontic treatment of maxillary molars can be performed with greater precision, improving long-term prognosis and reducing the likelihood of retreatment or extraction.

To address the clinical importance of anatomical variations in root canal morphology, the present study aims to conduct a systematic review and meta-analysis of the prevalence of MB2 canals in maxillary first and second molars detected using CBCT imaging in the Han population. Given the population-specific differences in dental anatomy reported in previous studies, a focused analysis on the Han ethnic group will provide regionally relevant and more accurate data to guide diagnosis and treatment strategies.^5^ By synthesizing available evidence, this review seeks to clarify the true prevalence of MB2 from a large number of participants in this population and contribute to the growing body of knowledge necessary for optimizing endodontic care.

## Materials and methods

This study was conducted consistent with the Preferred Reporting Items for Systematic Reviews and Meta-Analyses (PRISMA) checklist (Table S1) and registered in PROSPERO with the registration number (CRD420251135362).

### Database search and identify eligible manuscripts

A systematic article search was managed and retrieved from PubMed, MEDLINE Complete, Scopus, ClinicalKey and registry from initiation to August 17th, 2025, using the following strategy (“Maxillary molar” OR “Maxillary first molar” OR “Maxillary second molar”) AND (“China” OR “Chinese” OR “Taiwan” OR “Taiwanese” OR “Han”) AND (“CBCT”) AND (“second mesiobuccal canal” OR “MB2”). Manual searches were completed for possibly eligible publications from the *Journal of Endodontics*, *International Endodontic Journal* and *Australian Endodontic Journal*.

### Inclusion and exclusion

The aim of this systematic review and meta-analysis was to synthesize clinical studies reporting the prevalence of second mesiobuccal canals (MB2) in permanent maxillary first and/or second molars among individuals of Han ethnicity. The inclusion criteria were as follows:

1. Studies had to include participants from Taiwan, Hong Kong, mainland China, or other regions reporting on Han populations.
2. Articles must report the number of participants, number of evaluated teeth, number of teeth with MB2, or the prevalence of MB2 in permanent maxillary first and/or second molars.
3. *In vivo* studies using CBCT for MB2 detection were included.
4. Abstracts and full-text articles must be accessible and written in either English or Chinese.

Studies were excluded if they met any of the following criteria:

1. *Ex vivo* or *in vitro* studies, case reports, review articles, short communications, or letters to the editor.
2. Studies that did not disclose, or from which the number of participants and MB2 prevalence could not be determined.
3. If multiple articles used overlapping participant populations, only one publication was included in the analysis.

### Data extraction and quality evaluation

Two authors independently chosen articles that met the inclusion and exclusion criteria and extracted relevant data from the manuscripts. Any discrepancies between the two reviewers were resolved by the corresponding author. Published information, including the number of participants, the number of teeth examined, and the prevalence of MB2 canals in maxillary first or second molars, was retrieved. In cases where data were incomplete, corresponding authors were contacted via email to obtain the raw data. The quality of selected studies was assessed by the corresponding author using the Newcastle–Ottawa Scale (NOS). Any disagreements during the evaluation were also adjudicated by the corresponding author.

### Statistical analysis

Due to the heterogeneity of the target populations across the selected studies, this meta-analysis was conducted using a random-effects model.^6^ And implemented via Comprehensive Meta-Analysis software (version 4, Biostat, Englewood, NJ, USA). A two-tailed *P*-value of less than 0.05 was considered statistically significant. To estimate the primary outcome, the prevalence of MB2 canal, we calculated event rates along with their corresponding 95 % confidence intervals (CIs). For the secondary outcomes, including sex and side distributions, we computed odds ratios (ORs) and their 95 % CIs.

Heterogeneity among studies was assessed using both the *I*^2^ and Q statistics. *I*^2^ values of 25 %, 50 %, and 75 % were interpreted as representing low, moderate, and high heterogeneity, respectively.^7^ The Q statistic was used to test the null hypothesis that all included studies shared a common effect size. If this assumption holds, the expected Q value would equal the degrees of freedom (i.e., the number of studies minus one). Funnel plots were generated and visually examined for evidence of publication bias. Egger's regression test was performed when at least ten datasets were available.^8^

## Results

### Study identification and selection

**Figure 1:**
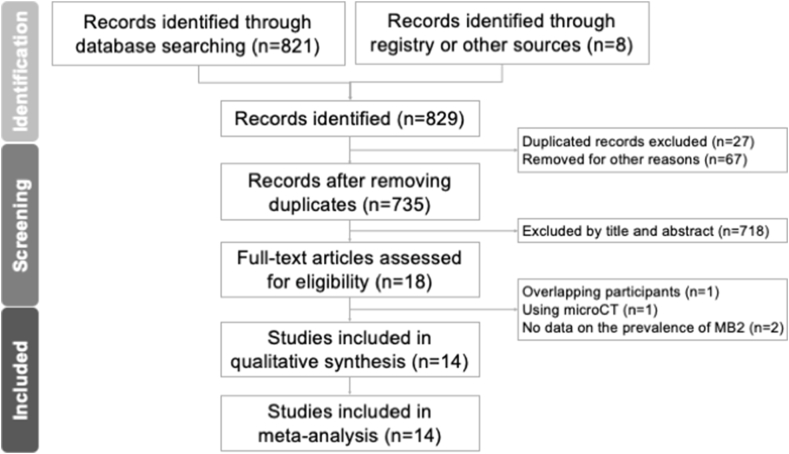
Preferred Reporting Items for Systematic Reviews and Meta-Analyses (PRISMA) flowchart of the selection process.

The PRISMA flowchart illustrating the literature selection process is presented in Fig. 1. After removing duplicate entries and excluding irrelevant studies based on titles and abstracts, a total of 14 studies met the inclusion criteria and were incorporated into the final analysis, 3 articles from Taiwan and 11 research from China. These eligible publications encompassed 15,639 participants, 35,929 teeth including 26,222 maxillary first and 9707 maxillary second molars, with participant ages ranging from 10 to 89 years. A detailed summary of the extracted data is provided in Table 1.^5^^,^^9^, ^10^, ^11^, ^12^, ^13^, ^14^, ^15^, ^16^, ^17^, ^18^, ^19^, ^20^, ^21^

**Figure 2:**
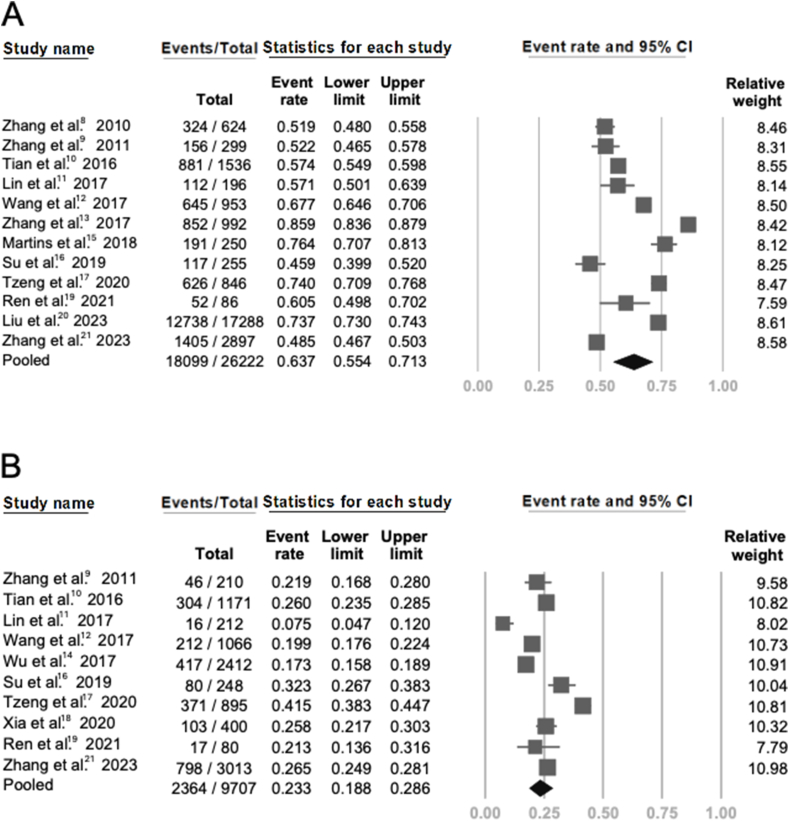
**Meta-analysis for included studies** (A) Forest plot of 12 studies about prevalence of second mesiobuccal canal (MB2) in permanent maxillary first molars (PMFMs). (B) Forest plot of 10 studies about prevalence of MB2 in permanent maxillary second molars (PMSMs).

**Table 1:** Characteristics of the final selected studies.

Study	Country	Patient N	Age range (mean ± SD)	Tooth type	Tooth N	Tooth N Gender (F/M)	Tooth N Side (L/R)
Zhang et al.^8^ 2010	China	548	10-86 (30.2 ± 14.5)	Maxillary first molar	624	(296/328)	(351/273)
Zhang et al.^9^ 2011	China	269	N/A	Maxillary first molar Maxillary second molar	299 210	N/A	N/A
Tian et al.^10^ 2016	China	844	14-81 (34.1 ± 15.1)	Maxillary first molar Maxillary second molar	1536 1171	N/A	N/A
Lin et al.^11^ 2017	Taiwan	114	18-64 (24.6 ± 13.2)	Maxillary first molar Maxillary second molar	196 212	N/A	N/A
Wang et al.^12^ 2017	China	647	18-80 (46.3 ± 14.2)	Maxillary first molar Maxillary second molar	953 1066	N/A	(477/476) (541/525)
Zhang et al.^13^ 2017	China	548	16-70 (33.4 ± 12.4)	Maxillary first molar	992	(465/527)	(493/499)
Wu et al.^14^ 2017	China	1294	20-78 (37.9 ± 14.2)	Maxillary second molar	2412	N/A	(1208/1204)
Martins et al.^15^ 2018	China	127	21-60 (34.3 ± 8.4)	Maxillary first molar	250	(132/118)	(125/125)
Su et al.^16^ 2019	Taiwan	216	(47 ± 14.6)	Maxillary first molar Maxillary second molar	255 248	(94/161) (98/150)	(129/126) (129/119)
Tzeng et al.^17^ 2020	Taiwan	519	18–65	Maxillary first molar Maxillary second molar	846 895	(463/383) (503/392)	(427/419) (453/442)
Xia et al.^18^ 2020	China	200	N/A	Maxillary second molar	400	(228/172)	(200/200)
Ren et al.^19^ 2021	China	50	16-55 (28.8 ± 7.5)	Maxillary first molar Maxillary second molar	86 80	(42/44) (39/41)	N/A
Liu et al.^20^ 2023	China	8644	10-89 (29.9 ± 12.1)	Maxillary first molar	17288	(11460/5828)	(8644/8644)
Zhang et al.^21^ 2023	China	1619	N/A	Maxillary first molar Maxillary second molar	2897 3013	(1814/1083) (1888/1125)	(1444/1453) (1506/1507)
Total		15639	10–89		35929		

N, number; F, female; M, male; L, left; R, right; N/A, not available.

### Heterogeneity and quality of the included studies

The *I*^*2*^ statistic was 99 % in PMFMs and 96 % in PMSMs, indicating that approximately 99 % and 96 % of the observed variability in effect sizes among studies was due to true heterogeneity rather than sampling error, respectively. The Q-statistic was used to assess the null hypothesis that all included studies share a common effect size. If this hypothesis were true, the Q-value would equal the degrees of freedom (number of studies minus one). In PMFM analysis, the Q-value was 1136.020 with 11 degrees of freedom (*P* < 0.001) and the Q-value was 255.223 with 9 degrees of freedom (*P* < 0.001) in PMSM investigation, suggesting significant heterogeneity of the included studies. At an alpha level of 0.10, the null hypothesis was rejected, confirming that the effect sizes varied across studies. Regarding methodological quality, 92.8 % of the studies were rated as high quality. One study was classified as moderate quality due to missing demographic data (age, sex, and side distribution). No studies were deemed to have a high risk of bias. A detailed summary of the quality assessments is provided in Table S2.

### Prevalence of MB2 in maxillary first and second molars

Among the 14 included studies, 12 reported the event rate (i.e., prevalence) of MB2 canals in PMFMs. Out of a total of 26,222 PMFMs examined, 18,099 teeth exhibited the presence of MB2, resulting in a pooled event rate of 0.637 (95 % CI = 0.554–0.713), indicating an overall MB2 prevalence of 63.7 %. Compared with previous Taiwanese studies, the pooled prevalence observed in this analysis was higher than that reported by Lin et al.^12^ and Su et al.,^16^ but lower than the value reported by Tzeng et al.^17^

Regarding PMSMs, data from 10 studies were analyzed, encompassing a total of 9707 teeth. Among these, 2364 teeth were identified with MB2 canals. The pooled event rate was 0.233 (95 % CI: 0.188–0.286), indicating an overall MB2 prevalence of 23.3 %. This estimate is notably higher than the 7.5 % reported by Lin et al.,^12^ but lower than the prevalence rates observed in the studies by Su et al.^16^ (32.3 %) and Tzeng et al.^17^ (41.5 %) (see Fig. 2).

### Prevalence of MB2 in maxillary first and second molars between male and female

**Figure 3:**
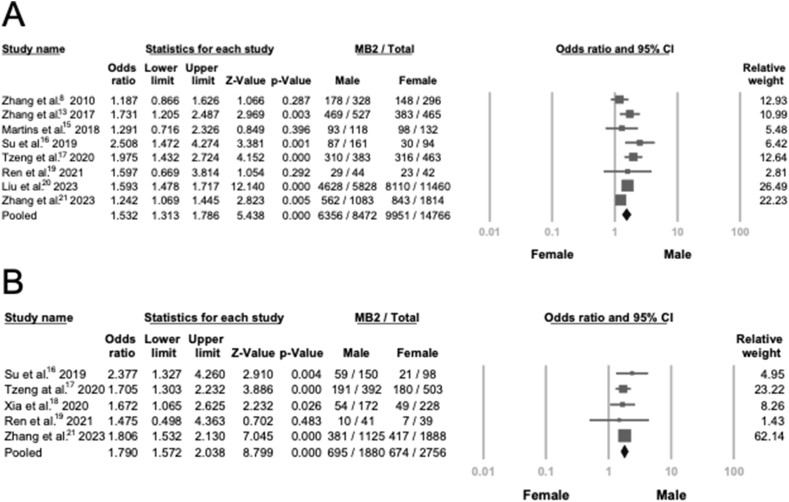
**Meta-analysis for included studies by sex** (A) Forest plot of 8 studies about prevalence of MB2 in PMFMs between male and female. (B) Forest plot of 5 studies about prevalence of MB2 in PMSMs between male and female.

We further examined the difference in MB2 prevalence between male and female participants. Among the included studies, 8 out of 14 reported detailed data on the tooth number of PMFMs from males and females, along with the corresponding number of MB2 canals in each subgroup. Similarly, 5 studies provided such data for PMSMs. For PMFMs, the pooled odds ratio (OR) was 1.532 with a *P* < 0.001 and a 95 % confidence interval (CI) of 1.313–1.786, indicating that males were 1.532 times more likely to have MB2 canals than females. A comparable trend was observed in PMSMs, where the pooled OR was 1.790 (95 % CI: 1.572–2.038, *P* < 0.001), suggesting that males also had higher odds of presenting MB2 canals in PMSMs. Detailed results are illustrated in Fig. 3. The publication bias was tested and the funnel plots showed symmetric distribution (Fig. S1 and S2).

### Prevalence of MB2 in maxillary first and second molars in different side

**Figure 4:**
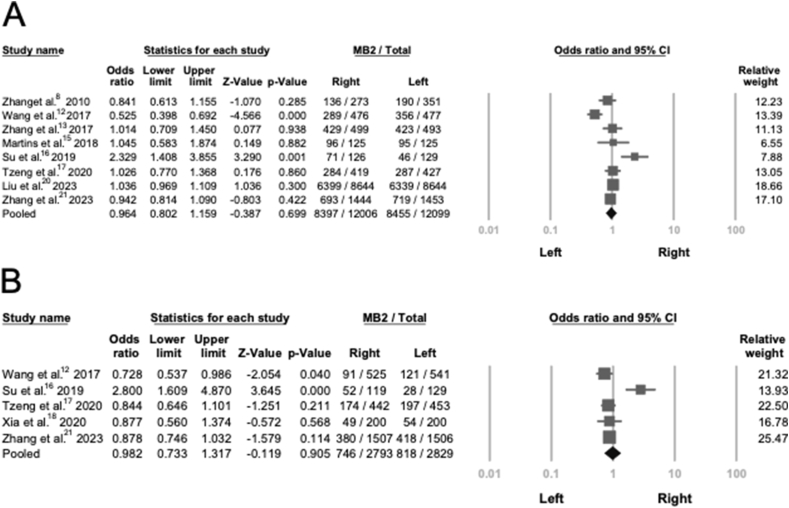
**Meta-analysis for included studies by tooth position** (A) Forest plot of 8 studies about prevalence of MB2 in PMFMs between left and right side. (B) Forest plot of 5 studies about prevalence of MB2 in PMSMs between left and right side.

Next, we evaluated the distribution of MB2 canals between the left and right sides, as some studies have suggested a positional preference. While certain reports indicated a higher prevalence on the right side,^16^ others suggested a dominance on the left.^13^ However, our pooled analysis revealed no statistically significant difference in MB2 prevalence between the left and right sides in either PMFMs (*P* = 0.699) or PMSMs (*P* = 0.905). As shown in Fig. 4, studies reporting a side preference generally included a smaller sample size. Funnel plot assessments are presented in Fig. S3 and S4.

## Discussion

This study aimed to investigate the prevalence of second mesiobuccal (MB2) canals in maxillary first and second molars (PMFMs and PMSMs) among the Han population, utilizing CBCT imaging. Our findings highlight the prevalence of MB2 canals in PMFMs (63.7 %) and the prevalence in PMSMs (23.3 %), with substantial heterogeneity observed across studies. It should be noted that this meta-analysis focused exclusively on studies reporting data from Han Chinese populations in regions such as Mainland China, Taiwan, and Hong Kong. The term “Chinese,” however, is broader and also includes overseas populations of Chinese descent, who may have different genetic backgrounds, lifestyles, and cultural habits due to intermarriage and regional influences. Therefore, our findings should be interpreted as specific to the Han Chinese population and may not be directly generalizable to the wider global Chinese dispersion. These results align with previous literature indicating the complex internal anatomy of maxillary molars and emphasize the clinical importance of recognizing MB2 canals for improving endodontic success rates.^22^ The high MB2 prevalence in PMFMs supports previous studies indicating that these molars commonly present with two canals in the mesiobuccal root.^14^^,^^20^ However, our pooled estimate slightly exceeds earlier reports from Taiwan,^12^^,^^16^ but remains lower than some individual Chinese studies.^14^^,^^20^ This discrepancy could stem from sample size differences, or variability in CBCT resolution and interpretation protocols. The prevalence in PMSMs, although significantly lower than that in PMFMs, still warrants clinical attention due to the anatomical challenges and the consequences of missed canals on treatment outcomes.

A notable sex-related difference in MB2 canal prevalence was identified.^14^^,^^16^^,^^17^^,^^20^^,^^21^ Males exhibited significantly higher odds of having MB2 canals in both PMFMs (OR = 1.532) and PMSMs (OR = 1.790) compared to females. This gender-related discrepancy may be attributed to anatomical differences in root canal morphology influenced by genetic factors. From a clinical perspective, these findings advocate for heightened vigilance during root canal treatment in male patients, particularly in maxillary molars, where MB2 canal detection and negotiation may be more likely. In contrast, the presence of MB2 canals showed no statistically significant difference between the left and right sides in either PMFMs or PMSMs. While individual studies have previously suggested a potential side dominance, our meta-analysis, incorporating larger pooled data, suggests this may not be a consistent or clinically relevant factor. Importantly, studies that reported lateral preferences often had smaller sample sizes, which may limit their generalizability.^16^ Heterogeneity among the included studies was substantial (*I*^*2*^ = 99 % for PMFMs and 96 % for PMSMs), reflecting diverse sample demographics, and imaging criteria. Despite this, the overall quality of the included studies was high, with 92.8 % meeting stringent methodological standards (Table S3). Only one study was classified as moderate quality due to missing demographic data.

The symmetric distribution in the funnel plots indicated no significant publication bias, further supporting the robustness of our findings (Fig. S1–S3). Although the funnel plots did not reveal significant publication bias and the random-effects model accounted for between-study variability (Fig. S1–S4), the extremely high heterogeneity observed in our analysis indicates that the pooled prevalence should be interpreted cautiously. This variability likely reflects differences in CBCT imaging parameters, study design, and population characteristics. Therefore, the pooled estimate provides only a general indication of prevalence, and subgroup analyses or stratified approaches are warranted to better capture the complexity of the underlying data.

From a clinical standpoint, these findings highlight the critical role of preoperative imaging and anatomical awareness in endodontic treatment planning. CBCT remains a valuable diagnostic adjunct for identifying complex canal anatomies, particularly MB2 canals, which may not be evident on conventional radiographs. Endodontists and general practitioners alike should be encouraged to consider advanced imaging modalities when treating maxillary molars to reduce the risk of missed canals and subsequent treatment failure.

Several limitations must be acknowledged in the present meta-analysis, we observed a high degree of heterogeneity among the included studies. This variability is likely attributable to several substantive differences, including CBCT voxel size, field of view, exposure settings, publication year, and patient characteristics. To better understand the implications of this heterogeneity, we conducted sub-group analyses by sex and tooth side. These analyses demonstrated that although the exact prevalence estimates varied across sub-groups, the overall trend consistently indicated a relatively high prevalence of MB2 canals in Han populations. Nevertheless, we acknowledge that the high level of heterogeneity limits the precision of the pooled prevalence and highlights the need for cautious interpretation. Future investigations employing standardized CBCT protocols and uniform reporting criteria are warranted to reduce variability and improve comparability across studies.

In conclusion, this study revealed the prevalence of second mesiobuccal (MB2) canals in PMFMs (63.7 %) and in PMSMs (23.3 %) within the Han population, as identified through CBCT imaging. Male individuals were significantly more likely to exhibit MB2 canals in both molar types, while no significant difference was observed between left and right sides. These findings emphasize the clinical relevance of thorough anatomical evaluation prior to endodontic treatment. Given the potential for undetected MB2 canals to compromise treatment outcomes, clinicians may consider the routine use of advanced imaging modalities, such as CBCT with small FOV in endodontic mode, to enhance diagnostic accuracy.

## Declaration of competing interest

The authors have no conflicts of interest relevant to this article.
